# Postoperative Pain and Incisional Hernia of Specimen Extraction Sites for Minimally Invasive Rectal Cancer Surgery: Comparison of Periumbilical Midline Incision Versus Pfannenstiel Incision

**DOI:** 10.3390/jcm14082697

**Published:** 2025-04-15

**Authors:** Yasuhiro Takeda, Keisuke Goto, Teppei Kamada, Tadashi Abe, Takafumi Nakano, Yasuhiro Takano, Masahisa Ohkuma, Makoto Kosuge, Ken Eto

**Affiliations:** Department of Surgery, The Jikei University School of Medicine, 3-19-18, Nishi-shimbashi, Minato-ku, Tokyo 105-8461, Japan; keisuke.gg.ms@gmail.com (K.G.); teppei0911show@yahoo.co.jp (T.K.); tadashi.abe.0925@gmail.com (T.A.); nakanon_0701@yahoo.co.jp (T.N.); ytakano1864@yahoo.co.jp (Y.T.); masa.ohkuma@jikei.ac.jp (M.O.); kosuge@jikei.ac.jp (M.K.); etoken@jikei.ac.jp (K.E.)

**Keywords:** Pfannenstiel incision, rectal cancer, minimal invasive surgery, incisional hernia

## Abstract

**Background**: Recent studies indicate that minimally invasive surgery is widely accepted as the optimal procedure for colorectal cancer. However, the ideal location of the specimen extraction site remains unclear. This study aimed to compare the conventional periumbilical midline incision with the Pfannenstiel incision for specimen extraction during minimally invasive surgery for rectal cancer. **Methods**: This retrospective cohort study included 76 patients who underwent minimally invasive surgery (double-stapling technique anastomosis) for rectal cancer between January 2022 and June 2023. The postoperative short- and mid-term outcomes were compared between the periumbilical midline incision and Pfannenstiel incision groups. **Results**: The patients’ backgrounds were comparable between the two groups. There were no significant differences in the surgical outcomes or short-term postoperative complications. The Pfannenstiel incision demonstrated advantages, including reduced postoperative pain at rest and during movement, and a lower incidence of incisional hernia (*p* = 0.038). **Conclusions**: The Pfannenstiel incision is a safe and effective option associated with reduced postoperative pain and a lower risk of incisional hernia. Therefore, it can serve as a useful alternative for specimen extraction during minimally invasive rectal cancer surgery.

## 1. Introduction

Colorectal cancer is the second most common cancer in Japan [1], and its morbidity and mortality rates are increasing globally [2]. Surgical treatment for resectable colorectal cancer has evolved from conventional laparotomy to minimally invasive surgery, aided by advancements in surgical devices and techniques [3,4]. Minimally invasive surgery is reported to be comparable or superior to conventional laparotomy in terms of the blood loss, postoperative wound pain, surgical site infection (SSI), respiratory complications, and length of hospital stay [5,6,7,8].

Incisional hernia is a common complication following abdominal surgery, with an incidence rate of 13–20% after midline laparotomy [9,10]. In addition, incisional hernia is associated with a decline in quality of life and significant re-operation costs [11,12]. Although minimally invasive surgery is expected to reduce the incidence of incisional hernia because of the smaller midline incisions, the incidence rate remains similar, with frequent symptoms such as pain and incarceration [13,14]. The patient-related risk factors for incisional hernia include advanced age, steroid use, diabetes, malnutrition, and obesity [15,16,17,18,19]. In addition, surgical factors, such as the closure method, SSI and specimen extraction sites, have been reported [20,21]. The periumbilical midline incision remains the most commonly used incision for specimen extraction in minimally invasive surgery. On the other hand, there is increasing evidence to suggest that off-midline extraction incisions may avoid incisional hernia [14,20,22]. The Pfannenstiel incision is a transverse incision in the lower abdomen that is widely used in urological and gynecological open surgery [23]. Additionally, it has cosmetic advantages and is associated with less postoperative pain, a lower incidence of SSI, and a reduced risk of incisional hernia [24,25]. Recently, the usefulness of the Pfannenstiel incision as a specimen extraction site for minimally invasive surgery has also been reported [26,27]. However, there is limited evidence regarding the safety and efficacy of the Pfannenstiel incision as a specimen extraction site for minimally invasive rectal surgery.

Therefore, this study aimed to compare the short-term outcomes and the incidence of incisional hernia between the conventional periumbilical midline incision (C) group and the Pfannenstiel incision (P) group to evaluate the optimal specimen extraction site for minimally invasive rectal cancer surgery.

## 2. Materials and Methods

### 2.1. Patients

This retrospective study was approved by our institutional review board (approval no. 30-249[9270]). Data were collected from prospectively maintained databases from January 2022 to July 2023 for all consecutive patients who underwent minimally invasive surgery for rectal cancer at Jikei University School of Medicine. The exclusion criteria were abdominal perineal resection and simultaneous resection of distant metastases. The patients received intravenous acetaminophen (1000 mg/dose, 15 mg/kg/dose for patients weighing less than 50 kg) every 6 h from POD 1 to 3, and epidural anesthesia was administered at the discretion of the anesthesiologist. Postoperative pain was evaluated using the numerical rating scale (NRS), graded from 0 to 10, where 0 = no pain and 10 = the worst imaginable pain.

The patient characteristics, clinicopathological data, surgical factors, treatment out-comes, postoperative pain (measured using the NRS at rest and upon movement from POD 1 to POD 7), and complications were retrospectively compared between the C and P groups.

### 2.2. Surgical Procedure: Pfannenstiel Incision

**Figure 1:**
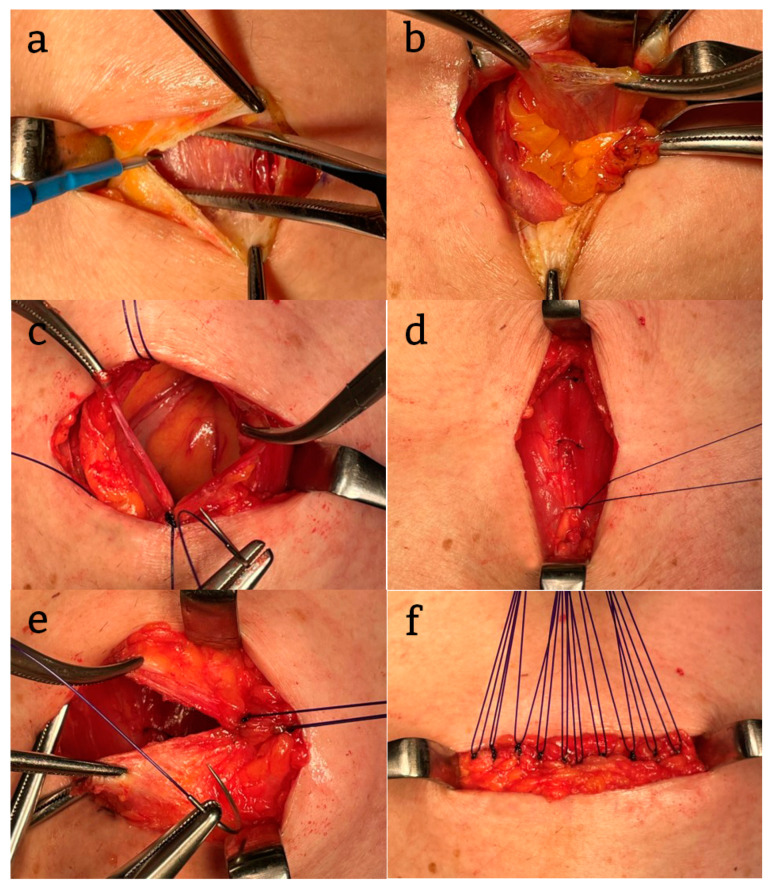
(**a**) The skin and anterior sheath of the rectus abdominis muscle were incised transversely above the pubic symphysis; (**b**) separation of the rectus abdominis muscle to the left and right and incision of the peritoneum longitudinally; (**c**) the peritoneum was closed longitudinally with continuous sutures; (**d**) the abdominis muscles was closed longitudinally with interrupted sutures; and (**e**,**f**) the anterior sheath of the rectus abdominis muscle was closed transversely with interrupted sutures.

A transverse incision of about 3–4 cm was made in the lower abdomen above the pubic symphysis, and the anterior sheath of the rectus abdominis muscle was also incised transversely (Figure 1a). The rectus abdominis muscle was bluntly separated to the left and right, and the peritoneum was incised longitudinally (Figure 1b).

After specimen extraction, the peritoneum was closed longitudinally with continuous sutures using absorbable thread (Figure 1c). The left and right rectus abdominis muscles were closed with interrupted sutures using absorbable thread (Figure 1d), and the anterior sheath of the rectus abdominis muscle was closed transversely with interrupted sutures using absorbable thread (Figure 1e,f). The skin was closed with buried sutures after washing the wound.

### 2.3. Follow-Up

The postoperative follow-up included a medical interview, physical examination, tumor marker testing every three months, and computed tomography (CT) or magnetic resonance imaging (MRI) every six months. Incisional hernia was diagnosed on the basis of imaging findings indicating a protrusion of visceral fat or part of the bowel above the level of the abdominal wall. When a hernia was suspected based on a medical interview and physical examination, CT or MRI was performed to confirm the diagnosis.

### 2.4. Statistical Analysis

The associations between each specimen’s extraction incision and the clinicopathological characteristics were analyzed using the Mann–Whitney U and Chi-Square (χ^2^) tests for continuous and categorical variables, respectively. The univariate analysis was performed using the χ^2^ test, and the multivariate analysis was performed using logistic regression analysis. The variables with *p* values < 0.10 in the univariate analysis were selected for the multivariate analysis. Statistical significance was set at *p* < 0.05. All the statistical analyses were performed using JMP^®^ 14 (SAS Institute Inc., Cary, NC, USA).

## 3. Results

### 3.1. Characteristics of the Study Cohort

Of the 76 patients included in this study, 30 were included in the C group and 46 were included in the P group. The clinicopathological characteristics of the two groups are listed in Table 1. The characteristics of the patients in the C and P groups were comparable, except for the frequency of robotic surgeries. In particular, the two groups were similar in terms of the preoperative nutritional status and diabetes.

### 3.2. Perioperative Outcomes

There were no significant differences between the two groups in terms of the surgical procedures, frequency of temporary stoma creation, or frequency and duration of epidural anesthesia. Overall, postoperative complications occurred in 10 patients (13.2%), among whom 2 (2.6%) had major complications of Clavien–Dindo grade ≥ IIIa. No significant difference was observed between the two groups in terms of the incidence of overall postoperative or major complications. Notably, no SSI cases were reported in the P group (Table 2).

### 3.3. Postoperative Pain

In terms of postoperative pain, the P group experienced less pain at rest on POD 1 (*p* = 0.032) and POD 7 (*p* = 0.019), and upon movement on POD 6 (*p* = 0.038) and POD 7 (*p* = 0.009) (Table 3 and Table 4). Furthermore, after excluding cases with epidural anesthesia, the postoperative pain was also better in the P group at rest on POD 3 (*p* = 0.020) and upon movement on POD 6 (*p* = 0.019) and POD 7 (*p* = 0.011) (Table 5 and Table 6).

### 3.4. Incisional Hernia

In both groups, the follow-up period was approximately two years, with no significant difference (*p* = 0.092). The P group had no incisional hernias, whereas the C group had an incidence rate of 15% (*p* = 0.038). In addition, the duration from surgery to the diagnosis of incisional hernia was approximately 10 months (Table 7). In the multivariate analysis, after accounting for multiple confounding factors, an advanced age (*p* = 0.022) and the specimen extraction sites (*p* = 0.011) were predictive of incisional hernia (Table S1).

## 4. Discussion

Conventionally, open surgery has been the standard treatment for resectable colorectal cancer. In recent years, minimally invasive surgery for colorectal cancer has become widely accepted, offering several advantages over open surgery [3,4,5,6,7,8]. Minimally invasive surgery for colorectal cancer requires a specimen extraction incision, and conventionally, a periumbilical midline incision that extends from the camera port in the navel has been used [28]. However, the optimal site for the specimen extraction incision for colorectal surgery remains unclear. Furthermore, some studies have indicated that the risk of postoperative complications, such as incisional hernia and SSI, is influenced by the specimen extraction site [14,20,22,28].

The Pfannenstiel incision, introduced by the German gynecologist Pfannenstiel, is a transverse lower abdominal incision [23]. It has been commonly used in gynecological and urological surgeries because it provides easy access to the deep pelvic organs [29,30,31]. This incision offers several benefits, including excellent cosmetic results due to following Langer’s line, reduced postoperative pain, and a lower incidence of SSI and incisional hernia [24,25]. The incidence of incisional hernia after open surgery is reported to be low at 0.5–2.1% [24,32] with the Pfannenstiel incision compared to 11–20% [9,10,33] with the midline incision. Furthermore, a meta-analysis comparing the specimen extraction sites in minimally invasive surgery reported that the risk of incisional hernia was significantly higher with a midline incision than with a non-midline incision [14,20,22,26,27]. The lower incidence of SSI and incisional hernia associated with the Pfannenstiel incisions may be attributed to (1) the lack of a continuous abdominal wall layer at the incision site and (2) avoidance of cutting the linea alba, which has the lowest blood flow in the abdominal wall [24,25]. Most cases of hernia occur within two years after surgery, although some occur later, emphasizing the importance of the follow-up period [33]. The follow-up period for both groups in this study was approximately two years. The incidence of incisional hernia was significantly lower in the P group, and no hernia cases were reported in this group.

Postoperative pain is associated with early mobilization, reduced respiratory complications, and a shorter hospital stay [34,35]. Although epidural anesthesia is effective in reducing postoperative incisional pain, its use is limited by its technical complexity and the potential risk of dural puncture. Alternatively, local anesthetic infiltration at the surgical site provides effective early analgesia; however, the short duration of analgesia presents a limitation [36]. Several reports have compared the postoperative pain between the Pfannenstiel and midline incisions in open abdominal or laparoscopic surgery; however, the results remain inconsistent [26,27,30,37]. In addition, there are few studies on postoperative pain at the specimen extraction sites after minimally invasive colorectal cancer surgery. In this study, the P group showed better postoperative pain outcomes at rest on POD 1 and 7, and upon movement on POD 6 and 7. There was no significant difference between the two groups in terms of the use of epidural anesthesia, and the duration of the epidural anesthesia use extended to POD 5 in both groups. When cases involving epidural anesthesia were excluded, the P group also showed better results at rest on POD 3 and POD 7, and upon movement on POD 6 and POD 7, suggesting that the Pfannenstiel incision was superior to the midline incision in terms of reducing the postoperative pain.

This study had several limitations. First, this was a single-center study with a limited number of patients. Second, as this was a retrospective study, the patient background and data collection could not be completely controlled. Although there were no significant differences in the clinicopathological characteristics between the two groups, except for the robotic surgery use, selection bias could not be ruled out. Finally, the choice of the incision type was left to the surgeon’s discretion. A prospective study with a larger sample size is needed to compare the benefits of the specimen extraction site in minimally invasive rectal surgery using the periumbilical midline incision and the Pfannenstiel incision.

## 5. Conclusions

The Pfannenstiel incision is preferable to the conventional periumbilical midline incision for minimally invasive rectal cancer surgery because it results in a lower incidence of incisional hernia and reduced postoperative pain.

## Figures and Tables

**Table 1:** Clinicopathological characteristics.

Variable	All (n = 76)	Conventional (n = 46)	Pfannenstiel (n = 30)	*p* Value
Gender, male, n (%)	47 (61.8)	27 (58.7)	20 (66.7)	0.630
Age, years, median (range)	62 (54–72)	65 (52–72)	60 (55–73)	0.894
BMI, kg/m^2^, median (range)	22.6 (20.8–25.2)	22.3 (20.5–25.1)	22.6 (20.9–25.6)	0.422
Albumin mg/mL, median (range)	4.3 (3.9–4.5)	4.3 (3.7–4.5)	4.2 (4.1–4.4)	0.840
ASA-PS, n (%)				0.719
1	12 (15.8)	6 (13.0)	6 (20.0)	
2	56 (73.7)	35 (76.1)	21 (70.0)	
3	8 (10.5)	5 (10.9)	3 (10.0)	
CCI, 2, n (%)	17 (22.4)	11 (23.9)	6 (20.0)	0.783
Age-adjusted ≥ CCI, 4, n (%)	35 (46.1)	22 (47.8)	13 (43.3)	0.815
History of abdominal surgery, yes, n (%) *	7 (9.2)	6 (13.0)	1 (3.3)	0.153
Diabetes, yes (%)	17 (22.4)	11 (23.9)	6 (20.0)	0.783
Neoadjuvant chemoradiotherapy, yes, n (%)	5 (6.9)	1 (2.2)	4 (13.8)	0.076
Surgical procedure, LAR, n (%)	36 (47.4)	18 (39.1)	18 (60.0)	0.101
Approach, robotic surgery, n (%)	26 (34.2)	11 (23.9)	15 (50.0)	0.026

Abbreviations: BMI, body mass index; ASA-PS, American Society of Anaesthesiologists Physical Status; CCI, Charlson comorbidity index. * Patients except for appendectomy and cesarean section.

**Table 2:** Short-term outcomes.

Variable	All (n = 76)	Conventional (n = 46)	Pfannenstiel (n = 30)	*p* Value
Operating time, ≥360 min, n (%)	36 (47.4)	18 (39.1)	18 (60.0)	0.101
Estimated blood loss, ≥50 mL, n (%)	14 (18.4)	6 (13.0)	8 (26.7)	0.225
Epidural anesthesia, yes (%)	36 (47.4)	20 (43.5)	16 (53.3)	0.483
Duration of epidural anesthesia, day *	3.8 ± 1.2	3.8 ± 1.4	3.9 ± 0.8	0.592
Pathological TNM stage				0.811
I–II	46 (60.5)	27 (58.7)	19 (63.3)	
III	30 (39.5)	19 (41.3)	11 (36.7)	
Adjuvant chemotherapy, yes, n (%)	29 (38.2)	21 (45.7)	8 (26.7)	0.147
Surgical site infection, n (%)	3 (4.0)	3 (6.5)	0 (0)	0.154
Postoperative complications, all (%)	10 (13.2)	8 (17.4)	2 (6.7)	0.299
Postoperative complication, ≥II (%)	6 (7.9)	5 (10.9)	1 (3.3)	0.234
Postoperative complication, ≥IIIa (%)	2 (2.6)	1 (2.2)	1 (3.3)	0.758
Mortality	0	0	0	1.000
Postoperative hospital stay, days (range)	14 (8–19)	13 (8–25)	14 (8–17)	0.533
Incisional hernia, yes, n (%)	7 (9.2)	7 (15.2)	0 (0)	0.038

* Mean ± SD.

**Table 3:** Postoperative numerical rating scale (NRS) at rest.

Postoperative Day	Conventional (n = 46)	Pfannenstiel (n = 30)	*p* Value
	
POD1	1.7 ± 1.4	1.0 ± 1.1	0.032
POD2	1.4 ± 1.4	0.9 ± 1.0	0.090
POD3	1.3 ± 1.9	0.6 ± 0.8	0.110
POD4	0.9 ± 1.2	0.6 ± 1.0	0.250
POD5	0.6 ± 1.0	0.5 ± 1.1	0.683
POD6	0.4 ± 0.8	0.3 ± 0.7	0.610
POD7	0.4 ± 0.6	0.1 ± 0.3	0.019

The data are presented as a mean ± SD.

**Table 4:** Postoperative numerical rating scale (NRS) upon movement.

Postoperative Day	Conventional (n = 46)	Pfannenstiel (n = 30)	*p* Value
	
POD1	3.9 ± 1.5	3.4 ± 2.3	0.342
POD2	4.0 ± 1.6	3.5 ± 2.2	0.296
POD3	3.5 ± 2.0	2.6 ± 1.4	0.087
POD4	2.5 ± 1.7	2.1 ± 1.6	0.194
POD5	2.3 ± 1.8	2.0 ± 1.7	0.414
POD6	1.9 ± 1.3	1.2 ± 1.3	0.038
POD7	1.4 ± 1.3	0.7 ± 1.2	0.009

The data are presented as a mean ± SD.

**Table 5:** Postoperative numerical rating scale (NRS) at rest, excluding patients with epidural anesthesia.

Postoperative Day	Conventional (n = 26)	Pfannenstiel (n = 14)	*p* Value
	
POD1	1.7 ± 1.5	0.9 ± 1.2	0.053
POD2	1.6 ± 1.5	0.7 ± 0.9	0.069
POD3	1.4 ± 2.0	0.3 ± 0.6	0.020
POD4	0.9 ± 1.1	0.5 ± 1.2	0.107
POD5	0.6 ± 0.8	0.5 ± 1.2	0.231
POD6	0.6 ± 0.8	0.4 ± 0.9	0.442
POD7	0.5 ± 0.7	0.1 ± 0.3	0.042

The data are presented as a mean ± SD.

**Table 6:** Postoperative numerical rating scale (NRS) upon movement, excluding patients with epidural anesthesia.

Postoperative Day	Conventional (n = 26)	Pfannenstiel (n = 14)	*p* Value
	
POD1	3.7 ± 1.5	3.5 ± 2.1	0.885
POD2	3.9 ± 1.5	3.4 ± 1.9	0.497
POD3	3.5 ± 2.2	2.4 ± 1.2	0.077
POD4	2.4 ± 1.1	1.9 ± 1.6	0.121
POD5	2.2 ± 1.5	2.0 ± 1.9	0.577
POD6	1.9 ± 1.2	0.9 ± 1.3	0.019
POD7	1.5 ± 1.2	0.5 ± 0.9	0.011

The data are presented as a mean ± SD.

**Table 7:** Incisional hernia.

Variable	Conventional (n = 46)	Pfannenstiel (n = 30)	*p* Value
	
Follow-up time, months *	24.5 ± 7.9	23.3 ± 3.8	0.092
Incisional hernia, n (%)	7 (15.2)	0 (0.0)	0.038
Time to hernia occurrence, months *	10.2 ± 2.3	N/A	

* Mean ± SD.

## Data Availability

The data presented in this study are available from the corresponding author upon reasonable request. The data are not publicly available since they are covered by institutional privacy policies.

## Supplementary Materials

The following supporting information can be downloaded at https://www.mdpi.com/article/10.3390/jcm14082697/s1, Table S1. Univariate and multivariate analyses of clinicopathological variables related to the incidence of incisional hernia.

## Abbreviations

The following abbreviations are used in this manuscript:

CT	Computed tomography
MRI	Magnetic resonance imaging
NRS	Numerical rating scale
SSI	Surgical site infection
