# Supplementary material for: Postoperative Pain and Incisional Hernia of Specimen Extraction Sites for Minimally Invasive Rectal Cancer Surgery: Comparison of Periumbilical Midline Incision Versus Pfannenstiel Incision
Source: J Clin Med. 2025 Apr 15;14(8):2697. doi: 10.3390/jcm14082697 (PMC12028115; doi:10.3390/jcm14082697)
Supplement: Supplementary file 1 [file jcm-14-02697-s001.zip › jcm-3506835-supplementary.pdf]

Table S1. Univariate and multivariate analysis of clinicopathological variables related to the incidence of incisional hernia

Variables	Univariate analysis		Multivariate analysis	
	OR (95% CI)	*P* value	OR (95% CI)	*P* value
Gender, female	4.69 (0,85-25.99)	0.098	6.52 (0.84-50.82)	0.054
Age, ≥ 65 years	8.28 (0.94-72.50)	0.044	11.38 (0.97-133.51)	0.022
BMI, ≥25 kg/m^2	4.08 (0.83–20.08)	0.087	5.57 (0.78–39.84)	0.078
Albumin, ≥ 4 mg/mL	2.49 (0.28-21.66)	0.666		
ASA-PS, ≥ 3	4.20 (0.67-26.48)	0.156		
CCI, ≥ 2	1.44 (0.25-8.18)	0.650		
Age-adjusted CCI, ≥ 4	3.25 (0.59-17.92)	0.238		
History of abdominal surgery, yes	1.75 (0.18-17.05)	0.507		
Diabetes, yes	1.44 (0.25–8.18)	0.650		
Neoadjuvant chemoradiotherapy, yes	NE*	0.461		
Surgical procedure, LAR	0.16 (0.02-1.42)	0.111		
Approach, robotic surgery	0.29 (0.03-2.58)	0.411		
Operating time, ≥ 360 min	0.41 (0.07-2.27)	0.435		
Estimated blood loss, ≥ 50 ml	0.72 (0.08-6.49)	0.767		
Specimen Extraction Sites, Conventional	NE*	0.038	NE*	0.011
Pathological TNM stage, I-II	1.71 (0.31-9.43)	0.697		
Adjuvant chemotherapy, yes	2.35 (0.49-11.34)	0.417		

Abbreviations: OR, odds ratio; CI, confidence interval; BMI, body mass index; ASA-PS, American society of Anaesthesiologists - Physical Status; CCI, charlson comorbidity index * NE = Not estimable, OR not estimable due to zero events in one group
